# CircRNAs in hepatic lipid metabolism: regulatory mechanisms and clinical implications

**DOI:** 10.3389/fgene.2026.1831613

**Published:** 2026-06-16

**Authors:** Yu Zeng, Meng Gao

**Affiliations:** School of Rehabilitation, Sichuan Vocational College of Health and Rehabilitation, Zigong, Sichuan, China

**Keywords:** biomarkers, circRNA, hepatic lipid metabolism, metabolic dysfunction-associated steatotic liver disease, molecular SPONGE, non-alcoholic fatty liver disease, signaling pathways

## Abstract

Hepatic lipid metabolism homeostasis is crucial for maintaining metabolic health, and its disruption is a central factor in the development of metabolic diseases such as non-alcoholic fatty liver disease (NAFLD) and metabolic dysfunction-associated steatotic liver disease (MASLD). Circular RNAs (circRNAs),a novel class of non-coding RNAs characterized by their covalently closed loop structures and remarkable stability, have emerged as key regulators of gene expression. Recent studies have revealed that circRNAs play significant roles in modulating critical hepatic lipid metabolism signaling pathways, including AMPK, mTOR, PPAR, and SREBP. This review systematically summarizes the latest advances in understanding how circRNAs influence these pathways through mechanisms such as acting as molecular sponges for microRNAs, interacting with proteins, and potentially encoding functional peptides. We critically evaluate the experimental models used in key studies, distinguishing between *in vitro*, *in vivo*, and clinical evidence, and discuss the context-dependent nature of circRNA function. Furthermore, the potential of circRNAs as diagnostic biomarkers and therapeutic targets for NAFLD and MASLD is discussed, highlighting their clinical relevance alongside a balanced assessment of the challenges facing clinical translation. By integrating current research findings, this review aims to provide a comprehensive theoretical foundation for elucidating the regulatory networks governing hepatic lipid metabolism and for developing innovative intervention strategies against metabolic liver diseases.

## 1 Introduction

The liver serves as the central organ for lipid metabolism, orchestrating a complex array of processes including fatty acid uptake, synthesis, oxidation, and lipoprotein secretion (Nguyen et al., 2008; Zhu et al., 2023). The precise regulation of lipid metabolic signaling pathways within hepatocytes is essential for maintaining lipid homeostasis (Yang et al., 2025). Disruption of these pathways leads to abnormal lipid accumulation in liver cells, which is a hallmark of nonalcoholic fatty liver disease (NAFLD) (Saiman et al., 2022; Teng et al., 2023). NAFLD encompasses a spectrum of liver conditions ranging from simple steatosis to nonalcoholic steatohepatitis (NASH),fibrosis, cirrhosis, and potentially hepatocellular carcinoma (HCC) (Anstee et al., 2019). Recently, the term metabolic dysfunction-associated steatotic liver disease (MASLD) has been proposed to replace NAFLD, reflecting a more precise definition of the disease spectrum. In this review, we primarily use “NAFLD” for consistency with the majority of the historical literature cited, but the terms are considered synonymous in the context of this discussion. The increasing prevalence of NAFLD worldwide underscores the urgent need to elucidate the molecular mechanisms governing hepatic lipid metabolism and its dysregulation (Foerster et al., 2022; Huang et al., 2020; Ioannou, 2021; Llovet et al., 2023). Recent advances have highlighted the critical involvement of noncoding RNAs (ncRNAs),particularly circular RNAs (circRNAs),in the regulation of lipid metabolic pathways and the pathogenesis of metabolic liver diseases (Duan et al., 2023; Safi et al., 2023; Zeng et al., 2021).

CircRNAs are a novel class of endogenous ncRNAs characterized by covalently closed loop structures formed through back-splicing of precursor mRNAs (Fu et al., 2021; Pisignano et al., 2023). This unique circular configuration confers remarkable stability, resistance to exonucleases, and tissue-specific expression patterns, distinguishing circRNAs from linear RNAs (Alzahrani, 2026). Functionally, circRNAs act as microRNA (miRNA) sponges, interact with RNA-binding proteins, modulate transcription, and in some cases, encode peptides (Li X. et al., 2025). It is important to note that the relative contribution of each mechanism is a subject of ongoing investigation, and many circRNAs may exert their primary functions through non-sponging activities. These multifaceted roles enable circRNAs to participate in diverse biological processes, including lipid metabolism, inflammation, and cellular stress responses, all of which are pivotal in liver physiology and disease (Yuan et al., 2022). Notably, circRNAs exhibit dynamic expression profiles during liver development and in response to metabolic challenges, suggesting their involvement in the fine-tuning of hepatic metabolic networks (Chen et al., 2022).

Emerging evidence from transcriptomic analyses across various species, including humans, mice, pigs, and geese, has revealed extensive circRNA expression in the liver, with many circRNAs differentially expressed during developmental stages or under pathological conditions such as obesity, NAFLD, and metabolic dysfunction-associated steatotic liver disease (MASLD) (Wang et al., 2025; Zhang et al., 2023; Wade et al., 2024). These studies have identified circRNAs that regulate key lipid metabolic pathways, including fatty acid biosynthesis, β-oxidation, and lipoprotein metabolism, often through competing endogenous RNA (ceRNA) networks involving circRNA-miRNA-mRNA interactions (Liu et al., 2024). For instance, *in vitro* and *in vivo* models have demonstrated that circRNAs have been shown to modulate the expression of genes involved in the peroxisome proliferator-activated receptor (PPAR) signaling pathway, AMP-activated protein kinase (AMPK) pathway, and sterol regulatory element-binding proteins (SREBPs),which are central regulators of lipid homeostasis (Huang et al., 2026; Miao et al., 2026). However, the consistency of circRNA expression profiles across different NAFLD models and human cohorts remains an area of active investigation, with some circRNAs showing model-dependent dysregulation.

The dysregulation of circRNAs contributes to the pathogenesis of NAFLD and its progression to more severe liver diseases (Miao et al., 2026). Specific circRNAs have been implicated in promoting hepatic lipid accumulation, oxidative stress, inflammation, and fibrosis. For example, studies in mouse models have shown that circSETD2 impairs hepatic lipid homeostasis by binding to carbamoyl phosphate synthetase 1 (CPS1),exacerbating lipid metabolic disturbances in MASLD (Xie et al., 2025). Similarly, circ-SLC9A6 encodes a novel peptide that promotes lipid dyshomeostasis via epigenetic regulation of CD36 transcription, a key fatty acid transporter, thereby contributing to NAFLD progression (Wang et al., 2024). Other circRNAs, such as circLDLR and circPTK2, have been reported in cell culture studies to alleviate lipid accumulation and steatosis by modulating autophagy and signaling pathways including SIRT1 and PI3K/Akt, highlighting their therapeutic potential (Yuan et al., 2022; Li et al., 2022). Moreover, circRNAs can influence the inflammatory milieu and oxidative stress in the liver, further impacting disease severity (Zhou et al., 2025).

Beyond their intracellular functions, circRNAs are enriched in exosomes—small extracellular vesicles that mediate intercellular communication—and can modulate systemic metabolic homeostasis and immune responses (Verwilt and Vromman, 2024). Exosomal circRNAs have been identified as pathogenic regulators and promising biomarkers in metabolic syndrome and liver diseases, including NAFLD and HCC (Taherpour et al., 2025; Xu et al., 2023). The stability and detectability of circRNAs in body fluids position them as attractive candidates for non-invasive diagnostic and prognostic biomarkers, as well as therapeutic targets (Zeng et al., 2023).

In addition to metabolic liver diseases, circRNAs play significant roles in liver fibrosis and hepatocellular carcinoma (Fu et al., 2021). They regulate hepatic stellate cell activation, extracellular matrix deposition, tumor proliferation, metastasis, and immune evasion through complex regulatory networks (Zhou et al., 2024; Liu et al., 2022; Li J. et al., 2024). The interplay between circRNAs and key signaling pathways such as TGF-β, Wnt/β-catenin, and MAPK further underscores their central role in liver pathophysiology (Bhat et al., 2024; Saleh et al., 2024; Samad et al., 2025; Zhang et al., 2024).

Collectively, these findings underscore the critical regulatory functions of circRNAs in hepatic lipid metabolism and liver disease progression. Understanding the mechanisms by which circRNAs modulate lipid metabolic signaling pathways offers novel insights into liver biology and disease, paving the way for the development of circRNA-based diagnostic tools and therapeutic interventions. This review aims to comprehensively summarize the current progress in circRNA research related to hepatic lipid metabolism, focusing on their mechanisms of action, involvement in specific signaling pathways, associations with liver diseases, and potential clinical applications. We also provide a critical appraisal of the experimental models used in the literature and discuss the key challenges that must be addressed for successful clinical translation.

## 2 Materials and methods: literature search Strategy

A systematic literature search was conducted to identify original research articles relevant to circRNAs in hepatic lipid metabolism and NAFLD/MASLD. The following databases were searched: PubMed, Web of Science, and Embase. The search was performed from database inception up to March 2024. The following keyword combinations were used: (“circular RNA” OR “circRNA”) AND (“lipid metabolism” OR “steatosis” OR “NAFLD” OR “MASLD” OR “NASH” OR “fatty liver”) AND (“liver” OR “hepatic”). Inclusion criteria were: (i) original peer-reviewed research articles; (ii) studies focusing on the role of circRNAs in hepatic lipid metabolism, NAFLD, MASLD, or NASH; (iii) articles published in English. Exclusion criteria were: reviews, meta-analyses, editorials, conference abstracts, and studies not directly involving liver-related lipid metabolic pathways. Reference lists of retrieved articles were also manually screened to identify additional relevant studies.

## 3 circRNA regulation mechanisms of hepatic lipid metabolism

### 3.1 Acting as miRNA “molecular sponges” to adsorb and regulate

Circular RNAs (circRNAs) have emerged as pivotal regulators of hepatic lipid metabolism primarily through their function as competing endogenous RNAs (ceRNAs),acting as molecular sponges that competitively bind specific microRNAs (miRNAs) via base complementarity (Ma et al., 2024). This interaction effectively sequesters miRNAs, thereby alleviating their inhibitory effects on target mRNAs and indirectly upregulating gene expression involved in lipid metabolic pathways. For instance, *in vitro* overexpression studies have shown that circRNA-0046367 can sponge miR-34a, a miRNA known to suppress peroxisome proliferator-activated receptor alpha (PPARα) mRNA.By adsorbing miR-34a, circRNA-0046367 releases PPARα from miRNA-mediated repression, enhancing fatty acid β-oxidation and mitigating hepatic lipid accumulation, thus reducing steatosis (Guo et al., 2017). It should be noted, however, that the functional significance of this sponging activity *in vivo* remains to be fully validated, and the expression level of circRNA-0046367 relative to miR-34a target sites warrants further quantitative assessment.

Recent discussions in the field have raised important considerations regarding the quantitative requirements for effective miRNA sponging. Many circRNAs are expressed at relatively low copy numbers, which may be insufficient to sequester a significant fraction of abundant miRNAs.In such cases, subcellular co-localization of circRNAs and their target miRNAs at specific functional sites (e.g.,P-bodies, stress granules) may facilitate effective competition despite low global abundance. Nonetheless, these quantitative concerns underscore the importance of investigating alternative mechanisms, such as protein binding and translational functions, which may represent the primary mode of action for many circRNAs.

Moreover, some circRNAs exhibit the capacity to bind multiple miRNAs simultaneously, forming intricate regulatory networks that fine-tune lipid homeostasis. Cell-based assays have demonstrated that circRNA-0001805 interacts with both miR-106a-5p and miR-20b-5p to regulate downstream genes implicated in lipid synthesis and transport, such as ABCA1 and CPT1, thereby influencing nonalcoholic fatty liver disease (NAFLD) progression (Li et al., 2021). The efficiency of this sponge effect is contingent upon several factors, including the abundance of circRNA expression, subcellular localization, and binding affinity to miRNAs.High expression levels and cytoplasmic localization favor effective miRNA sequestration, as observed in circHIPK3 and circACACA, which have been shown *in vitro* to modulate lipid metabolism by sponging miRNAs like miR-29a and miR-132b-5p, respectively (Li X. et al., 2025). Additionally, circRNAs such as circFASN have been implicated in regulating hepatic triglyceride homeostasis by sponging miR-33a, influencing sterol regulatory element-binding proteins (SREBPs) and lipid synthesis pathways, as demonstrated in cell culture models of post-transplant dyslipidemia (Zhang et al., 2020).

Experimental validation of miRNA sponging activity typically requires a combination of approaches, including: (i) Argonaute-2 RNA immunoprecipitation (AGO2-RIP) to confirm circRNA-miRNA binding in the RISC complex; (ii) luciferase reporter assays to demonstrate functional derepression of target mRNAs; and (iii) MS2-tagging systems to map circRNA-miRNA interactions. The absence of such comprehensive validation in some studies limits definitive conclusions about the prevalence of the sponging mechanism.

The circRNA-miRNA-mRNA axis is thus a fundamental regulatory mechanism in hepatic lipid metabolism, with circRNAs acting as molecular decoys that modulate gene expression networks governing fatty acid oxidation, lipogenesis, and cholesterol metabolism (Gao et al., 2025). This ceRNA mechanism not only elucidates the pathogenesis of metabolic liver diseases such as NAFLD and metabolic dysfunction-associated steatotic liver disease (MASLD) but also offers promising therapeutic targets (Gao et al., 2025). For example, *in vivo* overexpression of circZBTB46, in mouse models has been shown to alleviate MASLD by sponging miR-326 to upregulate FGF1, thereby reducing hepatic lipid accumulation (Zeng et al., 2026). Furthermore, nanodrug systems delivering circRNA_0001805 have been developed to overexpress this circRNA in hepatocytes, suppressing lipid metabolism disorder and inflammation via miR-106a-5p/miR-320a and ABCA1/CPT1 axes, demonstrating translational potential (Li et al., 2021).

### 3.2 Interaction with RNA-Binding proteins (RBPs)

Beyond their role as miRNA sponges, circRNAs modulate hepatic lipid metabolism through direct interactions with RNA-binding proteins (RBPs),serving as protein scaffolds or decoys that influence protein function, localization, and stability (Ma et al., 2024; Zhao et al., 2020; Cen et al., 2022). CircRNAs can bind specific RBPs to stabilize mRNAs encoding key lipid metabolic enzymes or to facilitate the assembly of protein complexes that regulate transcription of lipid-related genes. For example, studies in liver cell lines have shown that circRNA_0007334 interacts with the mRNA-binding protein of fatty acid synthase (FASN) in the liver, stabilizing FASN mRNA and promoting *de novo* fatty acid synthesis, thereby contributing to lipid accumulation (Yu et al., 2023). This interaction exemplifies how circRNAs can enhance the expression of lipogenic enzymes post-transcriptionally by modulating mRNA stability via RBP binding (Cen et al., 2022).

Additionally, circRNAs can act as platforms for assembling protein complexes that recruit transcription factors or epigenetic modifiers to promoters of lipid metabolism genes. Mouse model investigations have revealed that circSETD2, a circRNA upregulated in metabolic dysfunction-associated fatty liver disease (MAFLD),binds carbamoyl phosphate synthetase 1 (CPS1),an enzyme involved in nitrogen metabolism, reducing its enzymatic activity and exacerbating lipid metabolic disturbances (Xie et al., 2025). This interaction suggests that circRNAs can influence enzymatic activity and metabolic flux by direct protein binding. Moreover, the read-through circRNA RCRIN binds RPL8 protein to recruit the E3 ubiquitin ligase RNF2, promoting RPL8 degradation, which reduces ribosome numbers containing RPL8 and decreases lipid accumulation and endoplasmic reticulum stress in hepatocytes (Wang et al., 2025). This mechanism highlights circRNAs’ capacity to regulate protein turnover and ribosome heterogeneity, impacting lipid synthesis. The circRNA cVIM (mmu_circ_32994),derived from the vimentin gene, has been reported to promote hepatic stellate cell activation by sponging miR-122-5p and miR-9-5p, which in turn enhances TGF-β receptor expression and signaling, illustrating circRNA-mediated modulation of signaling pathways via RBP interactions (Zhou et al., 2024).

It is worth noting that many circRNA-RBP interactions have been identified through RNA pull-down assays coupled with mass spectrometry, and the functional consequences have been validated primarily in *in vitro* systems. The *in vivo* relevance of these interactions, particularly in the context of dynamic metabolic challenges, requires further investigation using animal models with tissue-specific manipulation of circRNA expression.

These examples underscore the multifaceted roles of circRNAs in hepatic lipid metabolism through RBP interactions, influencing mRNA stability, enzymatic activity, and transcriptional regulation. Such interactions expand the regulatory repertoire of circRNAs beyond miRNA sponging, providing additional layers of control over lipid metabolic pathways.

### 3.3 Potential translational functions and derived peptides

Although traditionally classified as noncoding RNAs, emerging evidence reveals that certain circRNAs possess internal ribosome entry sites (IRES) or N6-methyladenosine (m6A) modifications enabling their translation into functional peptides or proteins, which may directly regulate lipid metabolism enzymes or signaling pathways in the liver (Meyer et al., 2015; Ma et al., 2019; Nakashima et al., 2024). For example, analysis of human and mouse liver tissues has demonstrated that circ-SLC9A6, highly expressed in human and mouse liver during NAFLD development, encodes a novel peptide, SLC9A6-126aa, whose phosphorylation status modulates its subcellular localization and function. Unphosphorylated SLC9A6-126aa translocates to the nucleus under lipid overload, promoting CD36 transcription via MOF-mediated histone acetylation, thereby exacerbating lipid dyshomeostasis and NAFLD progression (Wang et al., 2024). This study provides a compelling example of a circRNA-derived peptide exerting direct epigenetic regulation.

Similarly, preliminary evidence suggests that circβ-catenin, a liver-specific circRNA, encodes a novel protein that modulates the Wnt/β-catenin signaling pathway, influencing hepatocyte proliferation and lipid metabolism (Guo et al., 2017). Moreover, circANKRD17 in pigs encodes a 571-amino-acid protein that activates the PPAR signaling pathway, promoting intramuscular fat metabolism by enhancing adipocyte differentiation, fatty acid transport, and triglyceride synthesis (He et al., 2025). The m6A modification plays a crucial role in regulating circRNA translation; for instance, YTHDF2-mediated degradation of m6A-modified circ-SLC9A6 controls peptide expression, linking epitranscriptomic regulation to circRNA function (Wang et al., 2024). However, the field of circRNA translation is still in its infancy, and the functional significance of most identified circRNA-encoded peptides remains to be rigorously validated *in vivo*.

These findings collectively suggest that circRNA-derived peptides can act as direct modulators of lipid metabolic enzymes or transcriptional regulators, influencing hepatic lipid homeostasis and disease progression. The translational potential of circRNAs thus represents an exciting frontier, offering novel biomarkers and therapeutic targets for metabolic liver diseases.

The majority of mechanistic studies on circRNAs in hepatic lipid metabolism have been conducted using *in vitro* cell culture models (e.g., HepG2, AML12 hepatocytes) or rodent models of diet-induced obesity and NAFLD. While these systems have provided invaluable insights into circRNA function, several limitations must be acknowledged. First, findings from immortalized cell lines may not fully recapitulate the complex metabolic milieu of primary hepatocytes or the intact liver. Second, high-fat diet-induced NAFLD models in mice exhibit variable penetrance and may not precisely mirror the chronic, multifactorial etiology of human disease. Third, inter-species differences in circRNA expression and function are well-documented; for instance, some circRNAs highly expressed in mouse liver have no clear human ortholog, and *vice versa*. Therefore, extrapolation of mechanistic findings to human physiology requires caution. Future studies should prioritize validation in primary human hepatocytes, liver organoids, and well-characterized human cohorts to strengthen translational relevance.

**FIGURE 1:**
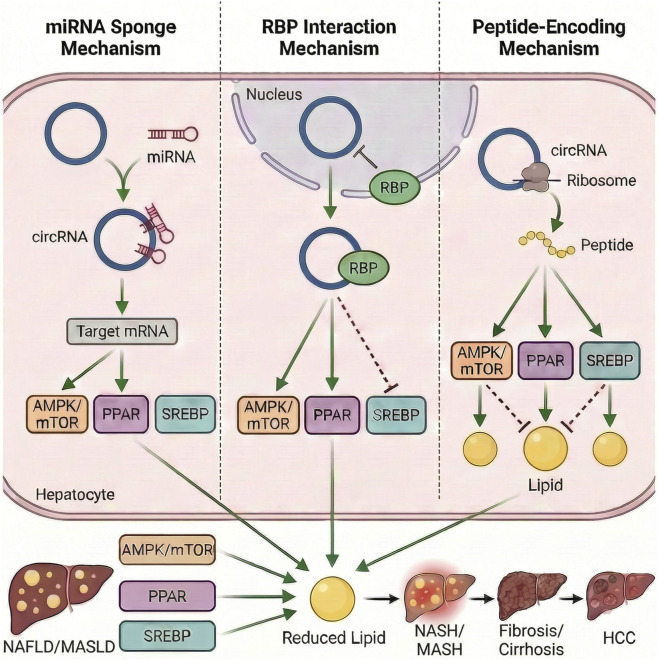
Three major mechanisms of circRNA regulation in hepatic lipid metabolism. The figure illustrates the three primary modes of action: **(A)** miRNA sponging, **(B)** RBP interaction, and **(C)** peptide translation, with representative examples for each mechanism.

We describe the three major molecular mechanisms underlying the circRNA-mediated regulation of hepatic lipid metabolism in Figure 1, including acting as miRNA molecular sponges, interacting with RNA-binding proteins (RBPs),and exerting translational functions to encode functional peptides.

## 4 circRNA-regulated key hepatic lipid metabolism signaling pathways

### 4.1 Regulation of AMPK/mTOR pathway

AMP-activated protein kinase (AMPK) serves as a critical cellular energy sensor that maintains energy homeostasis by inhibiting anabolic processes such as lipid synthesis and promoting catabolic pathways including fatty acid oxidation (Herzig and Shaw, 2017; Trefts and Shaw, 2021; Hardie et al., 2012). Activation of AMPK leads to suppression of sterol regulatory element-binding protein-1c (SREBP-1c)-mediated lipogenesis, thereby reducing hepatic lipid accumulation (Fang et al., 2022; Li et al., 2011). Recent studies have elucidated the role of circular RNAs (circRNAs) in modulating the AMPK/mTOR signaling axis, which is pivotal in hepatic lipid metabolism (Li J. et al., 2024; González et al., 2020; Li et al., 2019). For instance, *in vitro* and *in vivo* experiments have shown that circRNA_0075850 has been shown to act as a molecular sponge for miR-326-3p, thereby relieving its inhibitory effect on AMPKα1 expression. This upregulation of AMPKα1 activates the AMPK pathway, resulting in the suppression of SREBP-1c-driven fatty acid synthesis and enhancement of fatty acid oxidation, ultimately mitigating hepatic steatosis (Zeng et al., 2026). Conversely, gain and loss-of-function studies in hepatocytes indicate that circRNA_0004913 exerts an opposing effect by binding and inhibiting AMPK activity, which indirectly activates the mechanistic target of rapamycin complex 1 (mTORC1) signaling. mTORC1 is a key kinase promoting anabolic metabolism, including lipid biosynthesis. The activation of mTORC1 by circRNA_0004913 exacerbates lipid accumulation in the liver under high-fat diet conditions, highlighting the dual regulatory capacity of circRNAs in energy metabolism balance (Shao et al., 2023). However, the precise molecular details of how circRNA_0004913 inhibits AMPK activity remain to be fully elucidated.

This bidirectional modulation underscores the complexity of circRNA-mediated regulation of the AMPK/mTOR pathway in hepatic lipid homeostasis. Moreover, GLP-1 receptor agonists have been reported to induce weight loss partly through activation of the AMPK pathway via circRNA-associated competing endogenous RNA (ceRNA) networks, further emphasizing the therapeutic potential of targeting circRNA-AMPK/mTOR interactions in metabolic diseases (Li W. et al., 2025). Collectively, these findings demonstrate that circRNAs can fine-tune hepatic lipid metabolism by modulating the AMPK/mTOR signaling axis through miRNA sponging and direct interaction with pathway components.

### 4.2 Regulation of PPAR signaling pathway

Peroxisome proliferator-activated receptors (PPARs) are nuclear hormone receptors that play central roles in regulating lipid metabolism, including fatty acid uptake, storage, and oxidation (Li Y. et al., 2024). Among the PPAR isoforms, PPARγ primarily promotes lipid storage by facilitating fatty acid uptake and triglyceride accumulation in adipocytes and hepatocytes, whereas PPARα enhances fatty acid β-oxidation, thereby reducing lipid accumulation (Szántó et al., 2021). CircRNAs have emerged as important regulators of PPAR signaling in hepatic lipid metabolism. For example, cell culture studies demonstrate that circRNA_0128846 functions as a miRNA sponge for miR-127-5p, alleviating its repression on PPARγ expression. In fatty liver models, moderate upregulation of PPARγ via circRNA_0128846 promotes sequestration of fatty acids into relatively inert lipid droplets, exerting a protective effect against lipotoxicity (Liu et al., 2024). Similarly, *in vitro* overexpression of circRNA_0046367 enhances PPARα transcriptional activity through the miR-34a/PPARα axis, leading to increased expression of fatty acid oxidation genes such as carnitine palmitoyltransferase 1A (CPT1A). This circRNA-mediated activation of PPARα promotes lipid catabolism and mitigates hepatic steatosis, representing a prototypical example of circRNA-facilitated lipid breakdown (Guo et al., 2017). It should be noted that the protective effect of PPARγ upregulation is context-dependent and may vary across different stages of NAFLD.

These regulatory mechanisms highlight the capacity of circRNAs to modulate the balance between lipid storage and oxidation by targeting PPAR isoforms through ceRNA networks. Furthermore, the involvement of circRNAs in PPAR signaling pathways has been corroborated by transcriptomic analyses in various animal models and human liver tissues, underscoring their conserved and critical roles in lipid metabolism regulation (Chen et al., 2022). The modulation of PPAR activity by circRNAs offers promising avenues for therapeutic strategies aimed at restoring lipid homeostasis in metabolic liver diseases.

### 4.3 Regulation of SREBP-mediated lipid synthesis pathway

Sterol regulatory element-binding proteins (SREBPs),particularly SREBP-1c, are master transcription factors governing the expression of genes involved in fatty acid and cholesterol biosynthesis (Horton et al., 2002). Dysregulation of SREBP-1c activity leads to enhanced lipogenesis and contributes to hepatic steatosis. CircRNAs have been implicated in the fine regulation of SREBP-mediated lipid synthesis through multiple mechanisms. For instance, luciferase reporter and RNA pull-down assays have shown that circRNA_0000258 acts as a competing endogenous RNA by binding miR-130b-5p, thereby upregulating SREBP-1c expression and directly promoting triglyceride and cholesterol synthesis in the liver (Chen et al., 2023). Beyond miRNA sponging, certain circRNAs influence the post-translational processing of SREBPs.Mechanistic studies in colorectal cancer cells have revealed that circINSIG1 interacts with the SREBP cleavage-activating protein (SCAP),interfering with the transport of the SREBP-SCAP complex from the endoplasmic reticulum to the Golgi apparatus. This disruption inhibits SREBP maturation and activation, thereby suppressing lipid synthesis (Xiong et al., 2023). The relevance of this mechanism in hepatocytes requires dedicated investigation.

Such multifaceted regulation by circRNAs underscores their critical role in controlling hepatic lipid biosynthesis at both transcriptional and post-translational levels. Additionally, circRNAs modulating SREBP pathways have been linked to metabolic dysfunction-associated steatotic liver disease (MASLD) and nonalcoholic fatty liver disease (NAFLD),suggesting their potential as biomarkers and therapeutic targets.

Beyond the AMPK/mTOR, PPAR, and SREBP pathways, emerging evidence implicates circRNAs in the regulation of additional signaling cascades relevant to hepatic lipid metabolism and NAFLD pathogenesis. For example, circRNA_0057553 has been shown to modulate insulin signaling via the AKT pathway in hepatocytes, while other circRNAs have been linked to NF-κB-mediated inflammation and autophagy-related pathways. A comprehensive review of all pathways is beyond the scope of this article, but these examples illustrate the broader regulatory reach of circRNAs in liver biology.

We describe representative circRNAs that exert regulatory effects on the key signaling pathways of hepatic lipid metabolism, as well as their expression patterns, action mechanisms, target molecules, biological functions and relevant references in Table 1.

**TABLE 1:** Representative circRNAs Regulating Key Signaling Pathways in Hepatic Lipid Metabolism with Annotated Experimental Context.

circRNA name	Expression pattern	Mechanism of action	Target molecule(s)	Regulated pathway	Biological function	Experimental model	References
circRNA_0046367	Downregulated	miRNA sponge	miR-34a	PPARα	Promotes fatty acid β-oxidation, alleviates steatosis	HepG2 cells, mouse HFD model	Guo et al. (2017)
circRNA_0001805	Upregulated	miRNA sponge	miR-106a-5p, miR-320a	ABCA1/CPT1	Regulates cholesterol efflux and fatty acid oxidation	HepG2 cells, mouse NAFLD model	Li et al. (2021)
circSETD2	Upregulated	Protein binding	CPS1	Lipid metabolic enzyme activity	Exacerbates lipid metabolism disorders	Mouse MAFLD model	Xie et al. (2025)
circ-SLC9A6	Upregulated	Peptide encoding	CD36	Histone acetylation	Promotes lipid uptake, aggravates NAFLD	Human liver tissue, mouse NAFLD model	Wang et al. (2024)
circRNA_0001452	Downregulated	miRNA sponge	miR-466i-3p	AMPKα1	Activates AMPK, inhibits lipogenesis	HepG2 cells, mouse liver	Xie et al. (2022)
circRNA_0000258	Upregulated	miRNA sponge	miR-130b-5p	SREBP-1c	Promotes lipid synthesis	HepG2 cells, mouse HFD model	Liu et al. (2020)
circINSIG1	Downregulated	Protein binding	SCAP	SREBP processing	Inhibits SREBP maturation, reduces lipogenesis	CRC cells; hepatocyte relevance pending	Xiong et al. (2023)
circZBTB46	Downregulated	miRNA sponge	miR-326	FGF1/AMPK	Attenuates lipid accumulation	Mouse MASLD model	Zeng et al. (2026)

## 5 circRNA in the specific expression and function during NAFLD occurrence and development

### 5.1 Expression profile characteristics of circRNAs at different stages of NAFLD

High-throughput sequencing technologies have enabled comprehensive profiling of circular RNAs (circRNAs) across various stages of nonalcoholic fatty liver disease (NAFLD),revealing dynamic and stage-specific expression patterns in liver tissues and peripheral blood. Studies have demonstrated that circRNA expression profiles evolve from simple steatosis to nonalcoholic steatohepatitis (NASH) and further to liver fibrosis, reflecting the progressive pathological changes in NAFLD. For instance, circRNA_0070202 has been identified as significantly upregulated during the NASH stage, with its expression positively correlating with the degree of hepatic inflammation, suggesting a role in mediating inflammatory responses (Li et al., 2021). However, the reproducibility of these stage-specific signatures across independent patient cohorts remains to be systematically evaluated. Variability in patient demographics, disease etiology, and sequencing platforms may contribute to inconsistent findings.

These differentially expressed circRNAs are often enriched in pathways related to lipid metabolism, inflammation, and fibrogenesis, indicating their potential involvement in the pathophysiological mechanisms underlying NAFLD progression. Functional enrichment analyses, such as Gene Ontology (GO) and Kyoto Encyclopedia of Genes and Genomes (KEGG),consistently highlight circRNAs’ host genes participating in fatty acid biosynthesis, degradation, and PPAR signaling pathways, which are critical in hepatic lipid homeostasis and inflammatory regulation (Gao et al., 2025). Moreover, circRNA profiling in animal models, including high-fat diet-induced NAFLD mice and other species, has revealed circRNAs that modulate lipid accumulation and inflammatory signaling, further supporting their functional relevance (Wang et al., 2025). Peripheral blood circRNA signatures also reflect hepatic pathological changes, offering a minimally invasive window into disease staging. Nevertheless, the clinical utility of circulating circRNAs as stand-alone biomarkers requires validation in large, prospective studies with standardized detection methods.

### 5.2 Functional validation and mechanistic studies of key circRNAs

Functional investigations employing gain- and loss-of-function approaches have substantiated the regulatory roles of specific circRNAs in hepatic lipid metabolism and NAFLD pathogenesis. *In vitro* experiments using hepatocyte models demonstrate that silencing pro-lipogenic circRNAs, such as circRNA_0000258, significantly reduces intracellular lipid accumulation, indicating their facilitative role in steatosis (Li X. et al., 2025). Conversely, overexpression of anti-lipogenic circRNAs like circRNA_0046367 ameliorates lipid droplet formation induced by high-fat stimuli, highlighting their protective function against hepatic steatosis (Guo et al., 2017). These cellular findings are corroborated by *in vivo* studies utilizing transgenic mouse models with liver-specific circRNA overexpression or knockout. For example, circRNA_0057558 overexpression exacerbates high-fat diet-induced NAFLD phenotypes, including increased hepatic lipid deposition and inflammation, whereas circRNA_0046367 knockout mice exhibit aggravated steatosis and fibrosis, confirming their opposing roles in disease modulation (Wang et al., 2025). These gain and loss-of-function studies provide strong evidence for causal roles of specific circRNAs in NAFLD pathogenesis. However, the use of constitutive knockout or overexpression systems may not fully recapitulate the temporal dynamics of circRNA dysregulation during disease progression. Inducible, cell-type-specific genetic models are needed to dissect the precise functions of circRNAs at distinct disease stages and in specific liver cell populations.

Mechanistically, circRNAs act as competing endogenous RNAs (ceRNAs),sponging microRNAs that target key genes involved in lipid metabolism, inflammation, and fibrogenesis. For instance, circRNA_0001805 regulates NAFLD progression via the miR-106a-5p/miR-320a axis, modulating ABCA1 and CPT1 expression, which are critical for cholesterol efflux and fatty acid oxidation, respectively (Li et al., 2021). Similarly, circSETD2 impairs hepatic lipid homeostasis by binding to carbamoyl phosphate synthetase 1 (CPS1),thereby disrupting its enzymatic activity and exacerbating lipid metabolic disturbances (Xie et al., 2025). These mechanistic insights are further supported by circRNA-miRNA-mRNA network analyses and RNA immunoprecipitation assays, which delineate the intricate regulatory circuits. Collectively, the functional validation and mechanistic elucidation of key circRNAs affirm their pivotal roles in NAFLD pathophysiology and underscore their potential as therapeutic targets. The regulatory functions of circRNAs in NAFLD are highly context-dependent, varying by cell type and disease stage. For instance, circRNAs expressed in hepatocytes (e.g.,circRNA_0046367, circRNA_0000258) primarily influence lipid metabolic pathways, whereas those derived from hepatic stellate cells (e.g.,circRNA_0070202, cVIM) are more closely associated with fibrogenesis and extracellular matrix remodeling. Similarly, some circRNAs exhibit stage-specific dysregulation; for example, circRNA_0070202 is upregulated predominantly during the transition from steatosis to NASH, while others show altered expression early in disease progression. Understanding these spatiotemporal and cell-type-specific dynamics is essential for developing targeted therapeutic interventions and for interpreting biomarker data. Future studies should employ single-cell RNA sequencing and spatial transcriptomics to resolve circRNA expression at cellular resolution across the NAFLD spectrum.

We describe the expression profiles of key circRNAs at different stages of NAFLD, along with their sample sources, functional validation models and core research findings in Table 2.

**TABLE 2:** Summary of circRNA Expression Profiles and Functional Studies in Different Stages of NAFLD with Contextual Annotation.

circRNA name	Disease stage	Expression change	Sample source	Functional validation model	Primary cell type	Key findings	References
circRNA_0046367	Hepatic steatosis	Downregulated	Liver tissue	Cell/mouse	Hepatocyte	Overexpression alleviates lipid accumulation; protective circRNA	Guo et al. (2017)
circSETD2	MASLD	Upregulated	Liver tissue	Mouse	Hepatocyte	Binds CPS1, exacerbates lipid metabolism disorders	Xie et al. (2025)
circRNA_0001805	NAFLD	Downregulated	Liver tissue	Cell/nanodrug system	Hepatocyte	Overexpression improves lipid metabolism and inflammation	Li et al. (2021)
circLDLR	NAFLD	Downregulated	Liver tissue	Cell	Hepatocyte	Regulates autophagy via miR-667-5p/SIRT1 axis	Yuan et al. (2022)
circZBTB46	MASLD	Downregulated	Liver tissue	Mouse	Hepatocyte	Attenuates lipid accumulation via miR-326/FGF1 axis	Zeng et al. (2026)

## 6 circRNA as clinical biomarkers and therapeutic targets: potential and challenges

### 6.1 Development of diagnostic and prognostic biomarkers

Circular RNAs (circRNAs) have emerged as promising candidates for clinical biomarkers in liver diseases due to their remarkable stability in blood and exosomes, which facilitates their detection and quantification (Zhang et al., 2018; Verduci et al., 2021). Their covalently closed loop structure renders them resistant to exonuclease-mediated degradation, allowing circRNAs to persist in circulation and extracellular vesicles such as exosomes, making them accessible through minimally invasive liquid biopsies (Taherpour et al., 2025). For instance, specific circRNAs like circRNA_0070202 have been identified in serum, where their levels correlate significantly with hepatic fat content and inflammation scores, based on preliminary clinical cohort studies suggesting their utility as noninvasive adjunct markers for diagnosing nonalcoholic steatohepatitis (NASH) (Li et al., 2021). This is particularly valuable given the limitations of liver biopsy and the need for reliable biomarkers to stratify disease severity.

Moreover, combining multiple circRNAs into diagnostic panels enhances sensitivity and specificity beyond single markers or traditional clinical indices. For example, integrating circRNA_0001806 and circRNA_0128846 into composite models has improved discrimination between nonalcoholic fatty liver disease (NAFLD) stages, such as simple steatosis versus NASH, in retrospective analyses outperforming conventional biomarkers (Li et al., 2021). This multi-circRNA approach leverages the diverse regulatory roles of circRNAs in lipid metabolism and inflammation pathways, as evidenced by their involvement in competing endogenous RNA (ceRNA) networks that modulate gene expression relevant to disease progression (Zhang et al., 2023). Additionally, circRNAs such as circSETD2 and circZBTB46 have been shown to correlate with disease severity and prognosis in metabolic dysfunction-associated fatty liver disease (MASLD),further underscoring their potential as prognostic biomarkers (Xie et al., 2025; Zeng et al., 2026). The detection of circRNAs in exosomes also offers a window into the tumor microenvironment and systemic metabolic status, providing insights into disease mechanisms and therapeutic responses (Taherpour et al., 2025).

Despite this promise, several key challenges impede the clinical translation of circRNA biomarkers. First, there is a lack of standardized protocols for circRNA isolation, quantification, and normalization across different laboratories and platforms. Second, the specificity of many proposed circRNA biomarkers has not been rigorously tested against other liver diseases (e.g.,viral hepatitis, alcoholic liver disease) or metabolic conditions. Third, most studies have been conducted in relatively small, single-center cohorts, and large-scale, multi-center validation studies are urgently needed to establish clinical utility. Fourth, the dynamic changes in circRNA expression in response to therapeutic interventions and lifestyle modifications remain poorly characterized.

### 6.2 Prospects for therapeutic strategies targeting circRNAs

Therapeutic targeting of circRNAs represents a novel frontier in managing liver metabolic disorders, leveraging their regulatory capacity in lipid metabolism and fibrogenesis (Awata et al., 2025; Liao et al., 2025). Antisense oligonucleotides (ASOs) and small interfering RNAs (siRNAs) have been employed to specifically silence pathogenic circRNAs implicated in promoting hepatic steatosis and fibrosis. For example, preclinical studies in rodent models have shown that silencing of circRNAs that enhance lipogenesis or fibrogenic signaling can ameliorate fatty liver and attenuate fibrosis (Wang et al., 2025). The use of adeno-associated virus (AAV) vectors to deliver circRNAs with protective functions, such as circRNA_0046367, offers a gene therapy approach to restore beneficial circRNA expression and counteract disease progression (Guo et al., 2017). However, the unique circular structure of circRNAs poses technical challenges for efficient and specific artificial synthesis and *in vivo* overexpression, necessitating advances in vector design and delivery systems to achieve therapeutic levels without off-target effects.

## 7 Challenges and future directions for clinical translation

The clinical translation of circRNA-based therapeutics faces several formidable obstacles: (i) *In vivo* delivery and pharmacokinetics: Achieving efficient, tissue-specific delivery of circRNA-modulating agents (ASOs, siRNAs, or synthetic circRNAs) to hepatocytes while minimizing uptake by other organs remains a major hurdle. Nanoparticle-based carriers and engineered viral vectors show promise but require further optimization. (ii) Off-target effects: Given the complex ceRNA networks, modulating a single circRNA may inadvertently affect multiple miRNA-mRNA axes, leading to unforeseen consequences. Comprehensive off-target profiling and safety assessments are essential. (iii) Manufacturing and purity: Large-scale production of clinical-grade synthetic circRNAs with high purity and defined structural integrity is technically challenging and costly. (iv) Regulatory pathways: As a novel class of RNA therapeutics, circRNA-based drugs will require clear regulatory frameworks for approval, including standardized potency and safety assays.(v) Limited human data: To date, no circRNA-targeted therapy has entered clinical trials for liver disease, and all efficacy data derive from preclinical models.

Moreover, the complex regulatory networks involving circRNAs, microRNAs, and messenger RNAs exhibit functional redundancy and compensatory mechanisms, which may limit the efficacy of single-target interventions (Shao et al., 2023). Therefore, future therapeutic strategies may require multi-target approaches that modulate circRNA-miRNA-mRNA axes or combine circRNA targeting with existing pharmacotherapies such as peroxisome proliferator-activated receptor (PPAR) agonists to achieve synergistic benefits (Shao et al., 2023). Additionally, the development of circRNA-based therapeutics must address challenges related to delivery specificity, immunogenicity, and long-term safety. Despite these hurdles, the expanding understanding of circRNA biology and advances in nucleic acid therapeutics position circRNAs as promising targets for innovative treatments aimed at correcting dysregulated lipid metabolism and fibrosis in liver diseases.

## 8 Conclusion

The exploration of circRNAs in hepatic lipid metabolism and NAFLD has revealed their multifaceted roles as regulators of key signaling pathways, including AMPK/mTOR, PPAR, and SREBP. Through mechanisms such as miRNA sponging, RBP interaction, and peptide translation, circRNAs fine-tune fatty acid synthesis, oxidation, and transport, positioning them as pivotal modulators of metabolic homeostasis. However, the field must move beyond descriptive cataloging toward a deeper, context-aware understanding of circRNA function. Critical gaps remain in our knowledge of cell-type-specific actions, disease-stage dynamics, and species conservation. Moreover, the quantitative limitations of the miRNA sponge model necessitate rigorous experimental validation and a balanced consideration of alternative mechanisms.

From a translational perspective, circRNAs hold considerable promise as non-invasive biomarkers for NAFLD diagnosis and staging, yet their clinical utility hinges on standardization, large-scale validation, and demonstration of incremental value over existing tests. Therapeutically, circRNA modulation offers a novel avenue for intervention, but significant barriers in delivery, specificity, and safety must be surmounted before clinical application becomes feasible. Future research should prioritize the integration of advanced single-cell and spatial transcriptomics, the development of inducible and cell-type-specific genetic models, and the execution of well-powered clinical cohort studies. By addressing these challenges, the field can advance toward realizing the full potential of circRNAs in the diagnosis and treatment of metabolic liver diseases.
